# A systematic literature review and bayesian meta-analysis of oral vancomycin primary prophylaxis for *Clostridioides difficile* infection in stem cell transplant patients

**DOI:** 10.1017/ash.2025.10179

**Published:** 2025-10-14

**Authors:** Erika Viana-Cardenas, Eugenia Miranti, Wajeeha Tariq, Guillermo Rodriguez-Nava, Sa Shen, Evans Whitaker, Mingjun Jiang, Mindy M. Sampson, Andrew Rezvani, Ami S. Bhatt, Alexandre R. Marra, Jorge L. Salinas

**Affiliations:** 1 Division of Infectious Diseases and Geographic Medicine, Stanford University School of Medicine, Stanford, CA, USA; 2 Quantitative Sciences Unit, Stanford University, Stanford, CA, USA; 3 Lane Medical Library, Stanford University School of Medicine, Stanford, CA, USA; 4 Department Civil and Environmental Engineering, Stanford University, Stanford, CA, USA; 5 Division of Blood and Marrow Transplantation, Stanford University School of Medicine, Stanford, CA, USA; 6 Department of Medicine (Hematology) and of Genetics, Stanford University School of Medicine, Stanford, CA, USA; 7 Department of Internal Medicine, University of Iowa Carver College of Medicine, Iowa City, IA, USA; 8 Faculdade Israelita de Ciências da Saúde Albert Einstein, Hospital Israelita Albert Einstein, São Paulo, SP, Brazil

## Abstract

**Introduction:**

*Clostridioides difficile* infection (CDI) is common among patients undergoing hematopoietic stem cell transplantation (HSCT). Oral vancomycin prophylaxis may effectively prevent CDI in certain populations. We investigated the effectiveness of oral vancomycin primary prophylaxis in preventing CDI in HSCT patients.

**Methods:**

We searched six databases from inception to March 21, 2025, for studies comparing the incidence of CDI in HSCT patients who received oral vancomycin primary prophylaxis versus those who did not. We built a Bayesian random-effects model for meta-analysis. The primary outcome was the incidence of CDI. Secondary outcomes included incidence of positive vancomycin-resistant *Enterococcus* cultures, blood stream infections, graft-vs-host disease, and length of hospital stay. We also assessed for heterogeneity and publication bias using Robust Bayesian Meta-Analyses.

**Results:**

Six studies met inclusion criteria with a total of 1,236 patients. Four of the studies were of fair to good quality. Oral vancomycin primary prophylaxis reduced the incidence of CDI during hospitalization (OR 0.31; 95%CrI 0.16–0.59). Studies were weakly heterogeneous but had strong publication bias. Oral vancomycin primary prophylaxis reduced the odds of CDI by 12% after accounting for publication bias (OR 0.88; 95%CrI 0.32–1.16), although this reduction was not statistically significant. Secondary outcomes were similar in both groups.

**Conclusion:**

Oral vancomycin primary prophylaxis prevented CDI in HSCT patients without significantly affecting secondary outcomes. However, after controlling for publication bias, these findings were no longer significant. Further studies are needed to provide stronger evidence for or against this intervention, assess long-term safety, and assess potential effects on antimicrobial resistance.

## Introduction

*Clostridioides difficile* infection (CDI) is common and consequential among patients undergoing hematopoietic stem cell transplantation (HSCT), particularly allogeneic hematopoietic stem cell transplant recipients.^1^ Estimates of incidence of CDI in the first-year posttransplant range from ∼15–30%.^2,3^ The development of CDI in this population has been associated with an increased risk of graft-versus-host disease (GVHD) and mortality.^4,5^ Colonization with *C. difficile* is also prevalent among HSCT patients, with rates ranging from 9.3% to 29%.^6–8^ Colonization is associated with a higher risk for progression to CDI—which may be mitigated by providing prophylaxis to colonized patients.^9^

Several studies have indicated that oral vancomycin prophylaxis may effectively prevent CDI, particularly when used as secondary prophylaxis.^10–12^ There is great interest in whether oral vancomycin may also prevent CDI in the HSCT population, given their particularly high rates of CDI. However, there is also concern that oral vancomycin prophylaxis might lead to deleterious secondary outcomes such as vancomycin-resistant *Enterococcus* infection. To date, observational studies have not identified an impact of oral vancomycin prophylaxis on vancomycin-resistant *Enterococcus* infection rate.^13–17^ In the immunocompromised HSCT population, there is the additional risk for GVHD and in increased risk for bloodstream infection.^18^ Both could potentially be the result of alterations in the microbiome by oral vancomycin prophylaxis.

We aimed to investigate the effectiveness of oral vancomycin primary prophylaxis in preventing CDI in HSCT patients. By analyzing data from multiple studies, we sought to 1) provide a clearer understanding of the impact of oral vancomycin prophylaxis on CDI incidence, and 2) explore the impact of oral vancomycin prophylaxis on GVHD, vancomycin-resistant *Enterococcus* infection risk, bloodstream infection risk, and length of hospital stay. Our findings will contribute to the growing body of evidence evaluating the use of vancomycin as a prophylactic measure in the high-risk HSCT population.

## Methods

### Systematic literature review and search strategy

This systematic literature review was carried out following the guidelines set forth by the Preferred Reporting Items for Systematic Reviews and Meta-Analyses (PRISMA),^19^ and the Bayesian Analysis Reporting Guidelines (BARG).^20^ The review was registered with the International Prospective Register of Systematic Reviews (PROSPERO) on March 21, 2025 (CRD420251016925), with its protocol included. Approval from the Institutional Review Board was not necessary for this study and no patient informed consent was required either.

Our search strategy was developed in March 2025 with the assistance of a health sciences librarian. We explored Embase, PubMed, Scopus, Web of Science, Cochrane, and CINAHL. The literature search included manuscripts published from the inception of each database up to May 21, 2025. The detailed search strategy is available in the Supplementary Table 1.

Studies were included if they evaluated the incidence of CDI in patients who received oral vancomycin prophylaxis compared to those who did not. CDI was defined as detection of a positive nucleic acid amplification testing (NAAT) and/or reflex to toxin enzyme-linked immunosorbent assay for *C. difficile*, ideally in a two-step testing, in patients with new onset diarrhea (24 h or less).^9^ Primary prophylaxis was defined as the use of a drug to prevent disease in at-risk individuals with no prior history, while secondary prophylaxis aimed to prevent the recurrence of a disease in patients who have already experienced it. The intervention was defined as providing oral Vancomycin 125 mg twice daily, starting on the day of inpatient admission and continued until discharge. We excluded comments or reviews, studies that focused on secondary CDI, studies without a comparable control group, studies where controls received a different intervention (other than standard of care), pilot studies, and studies that used the same hospital population of an already included study.

We identified a total of 164 studies from our literature search. After removing duplicates, 132 studies were screened using title and abstract by two independent reviewers (WT and EV). From this initial review, 23 full-text studies were independently assessed (WT and EV). Discrepancies were resolved through discussion or consultation with a third reviewer (EM). Ultimately, six studies met inclusion criteria and were included in this systematic review.

### Data abstraction form and quality assessment

Two independent reviewers (WT, EM) abstracted data for each article using a standardized abstraction form. We recorded data regarding authors, publication year, study period, design, population selection, setting, inclusion and exclusion criteria of each study, studied groups, prophylaxis regimen, CDI diagnostic criteria, number of participants, total number of participants receiving the intervention, number of CDI per group, and secondary outcomes analyzed. Our primary outcome was the incidence of CDI in patients who received oral vancomycin prophylaxis and those who did not. Our secondary outcomes were bloodstream infections, vancomycin-resistant *Enterococcus* cultures, GVHD, and length of hospital stay.

We used the Downs and Black scale to assess quality of studies, which is specifically designed for randomized and non-randomized studies.^21^ All questions of the original published scale were answered for each reviewed paper and the total score calculated. We adapted question 27 of the Downs and Black scale replacing the multiple-choices options with a yes/no answer. The maximum possible score was 28. Downs and Black score ranges were given corresponding quality levels: excellent (26–28); good (20–25); fair (15–19); and poor (≤14). The reviewers (WT, EM, EV) performed the quality analysis independently and inconsistencies were resolved by discussion.

### Statistical analysis

Effect sizes and their standard errors were calculated from the sample sizes and reported cases for each study using the *metafor* package in R (version 4.8-0). The meta-analysis was conducted with the *bayesmeta* package version 3.4, employing Bayesian random-effects models.^22^ The Bayesian approach is especially useful to account for between-study heterogeneity. Additionally, unlike frequentist methods, it does not require a larger number of studies and directly provides credible intervals for the pooled mean effect. Half-normal prior was applied for both the overall effect and heterogeneity parameters. Heterogeneity is calculated using tau (τ), an estimate of the amount of true variability between the effect sizes of the included studies in a random-effects meta-analysis model. Results were summarized by reporting the posterior mean as an odds ratio (OR), along with 95% credible intervals (95% CrI) for the overall effect size and the tau statistic to quantify heterogeneity among the studies. And the prediction describes the consistency of data and model by comparing the actual data to data sets predicted via the posterior distribution.^22^

The potential for publication bias was assessed using funnel plot, Egger’s test, and the Robust Bayesian Meta-Analyses (RoBMA) package version 3.4.0.^23^ RoBMA results use Bayes factors (BF), a continuous measure of evidence in favor of the presence or absence of effect, heterogeneity, and publication bias. Bayes factor values above 10 indicate very strong evidence, from 3 to 10 moderate evidence, and from 1 to 3 weak evidence and <1 no evidence.^24^ Sensitivity analyses assessed the robustness of findings by excluding studies with unpublished full-text manuscripts. All analyses were performed using R version 2024.12.0 + 467.

## Results

### Study characteristics

**Figure 1.**
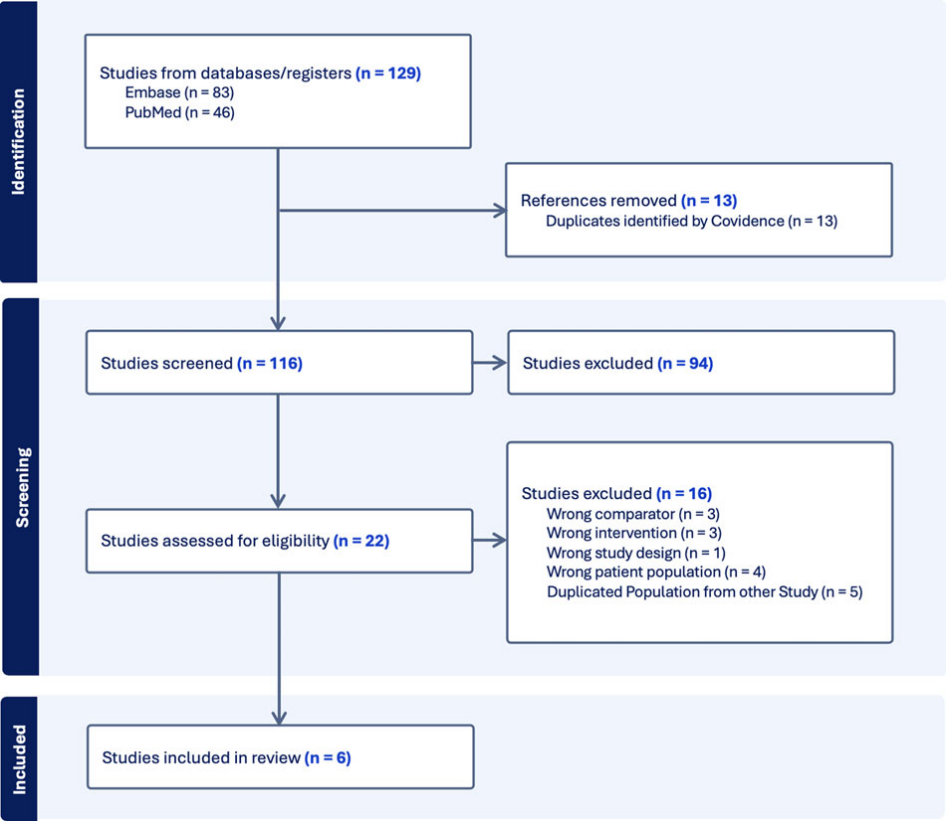
Literature search for studies published up to March 21, 2025, that evaluated the impact of oral Vancomycin prophylaxis for *Clostridioides difficile* infection in stem cell transplant patients.

From the 164 studies identified on the search strategy, a total of six met inclusion criteria to be in this systematic literature review and meta-analysis (Figure 1 and Table 1). Of them, four were studies published in full-text,^25–28^ and two were published abstracts.^29,30^ Five of them were conducted in academic medical centers, and one in a community hospital.^28^ All were conducted in the United States. Studies were performed between January 2012 and May 2023, and their duration varied from 11 to 120 months. In all six studies, patients received oral vancomycin 125 mg twice daily, starting on the day of inpatient admission and continued until discharge.

**Table 1.** Summary of study characteristics

First author, year, location Study period	Setting	Inclusion Criteria	Exclusion criteria	Studied Groups	Hospital Length of Stay in days	Prophylaxis Regimen	CDI Diagnostic Criteria	No. of Participants	Total CDI	Secondary Outcomes	D & B Score
Altemeier, 2022, Missouri, USA May 2017—Jan 2019	Academic medical center	Adults undergoing alloHSCT who were admitted on day 0	Active CDI at day 0; no oral vancomycin on day 0; OVP not continued for 7 days	OVP between March 2018 and January 2019 vs no OVP May 2017 to March 2018	Mean (SD) Intervention: 26.5 (14.1) Control 25.6 (11.2)	Oral Vancomycin 125 mg twice daily	Greater than three unformed stools in 1 day and positive detection of toxin B by PCR of stool sample.	100 OVP, 100 no OVP	CDI during hospitalization: 2/100 in OVP, 11/100 in no OVP	1) Rate of acute grades 2 – 4 graft vs host disease at day 100 2) Event free survival at 1 year	18
Ganetsky, 2019, Pennsylvania, USA April 2015—Nov 2016	Academic medical center	Adults undergoing alloHSCT	N/A	OVP between December 2015 to November 2016 vs no OVP April 2015 to December 2015	Median 29 vs 28 days; *P* = .22	Oral Vancomycin 125 mg twice daily	Commercial enzyme immunoassay used for Toxin A and B and glutamate dehydrogenase. If toxin was negative and glutamate dehydrogenase positive, PCR was performed for toxin genes.	90 OVP, 55 no OVP	CDI during hospitalization: 0/90 in OVP, 11/55 in no OVP	1) Acute, grades 2 – 4 GVHD 2) Acute, grades 3 – 4 GVHD 3) Chronic GVHD 4) Relapse 5) Non-relapse mortality 6) GVHD-free 7) relapse-free survival 8) overall survival	19
Ogawa, 2022, California, USA Not reported - April 2022	Academic medical center	AlloHSCT	N/A	OVP between June 2020 to April 2022 vs consecutive no OVP prior	N/A	Oral Vancomycin twice daily (Dose was not reported)	N/A	20 OVP, 20 no OVP	CDI at 100 days posttransplant: 0/20 in OVP, 5/20 in no OVP	1) VRE infection 2) Incidence of acute GVHD	15
Reitmeyer, 2024, New Jersey, USA Jan 2015—Aug 2022	Academic medical center	Adults at the time of admission for HSCT	Admitted for <72 hours; being treated or active CDI prior to day 0 of HSCT	OVP between December 2019 to August 31, 2022, vs no OVP between January 1 2015 to December 2019	Median (IQR) Control: 20 (16 – 24) Intervention: 21 (17 – 25) *P* value .5420	Oral Vancomycin 125 mg twice daily	A toxin/glutamate dehydrogenase was performed first if both were positive CDI was diagnosed. If inconsistent, test would reflex to PCR. If PCR was positive, CDI was diagnosed.	81 OVP, 75 no OVP	CDI during hospitalization: 1/81 OVP, 8/75 no OVP	1) Incidence of VRE infections 2) VRE isolated from any clinical culture 3) g bloodstream infections, 4) Hospital survival, 5) Hospital length of stay.	20
	Academic medical center	Admitted for HSCT	N/A	OVP vs no OVP. Not reported when Intervention started	N/A	Oral Vancomycin 125 mg twice daily	N/A	181 OVP, 260 no OVP	CDI during hospitalization: 13/181 OVP, 34/260 no OVP	1) Bacterial infectious complication 2) Transplant outcomes	13
Williams, 2024, Wisconsin, USA Jan 2012—Dec 2021	Community hospital	AutoHSCT for multiple myeloma or lymphoma	Stem cell transplant prior to study; >1 stem cell transplant in study period; alloHSCT or CAR T-cell therapy at any time	OVP (from Jan 2017) vs no OVP	Median (range) Overall 18 (12 – 37) Control: 19 (12 – 37) Intervention: 17 (12 – 37) *P*-value .003	Oral Vancomycin 125 mg twice daily	New onset diarrhea ( >3 loose stool in 24 hrs). Prior to 2018: PCR detection of cytotoxin B gene (positive test means CDI). After: reflex detection of glutamate dehydrogenase and toxins A and B added to PCR positive (positive EIA means CDI).	148 OVP, 106 no OVP	CDI during hospitalization: 6/148 OVP, 12/106 no OVP CDI during post discharge follow-up (till 180 days): 6/148 OVP, 2/106 no OVP	1) CDI incidence between groups in the postdischarge 180-day follow-up period 2) Differences in AutoHSCT admission length of stay	18

Abbreviations: AlloHCT, allogenic hematopoietic stem cell transplantation; AutoHSCT, autologous hematopoietic stem cell transplantation; CAR T-cell therapy, chimeric antigen receptor T-cell therapy; CDI, *Clostridioides difficile* infection; EIA, enzyme immunoassay; GVHD, graft-versus-host disease; HSCT, hematopoietic stem cell transplantation; OVP, oral vancomycin prophylaxis; PCR, polymerase chain reaction; N/A, Not available; VRE, vancomycin-resistant *Enterococci.*

All included studies were quasi-experimental: they conducted retrospective chart reviews, gathering data from both control and intervention populations before and after implementing the intervention. The intervention group included all patients admitted to the Bone Marrow Transplant unit after the implementation of oral vancomycin prophylaxis for HSCT. By contrast, the control group consisted of all patients from the same unit who underwent stem cell transplantation before the prophylaxis was introduced and did not receive this treatment.

All six studies reported the total number of participants (*n* = 1,236) with and without CDI as their primary outcome. The definition of CDI was explicitly discussed in five studies, while one study did not provide this information.^29^ Four studies employed a two-step diagnostic algorithm that combined immunoassay and NAAT for diagnosis, whereas one study utilized only NAAT.^25^ Two studies specified that testing was conducted for patients exhibiting clinical symptoms, defined as having more than three unformed stools within a 24-hour period,^25,28^ while one study indicated that testing was performed based on compatible clinical presentations.^27^ Notably, none of the studies assessed *C. difficile* colonization at baseline.

### Outcomes

**Figure 2.**
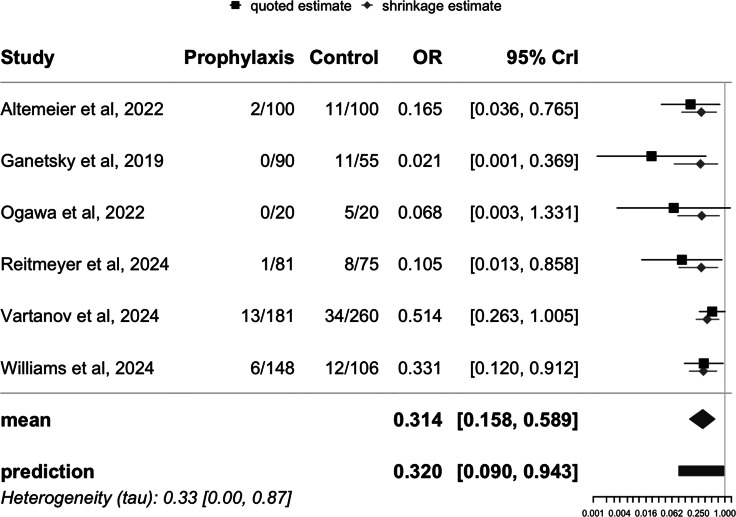
Forest plot of unadjusted *Clostridioides difficile* infection risk in stem cell transplant patients: impact of oral Vancomycin primary prophylaxis.

**Figure 3.**
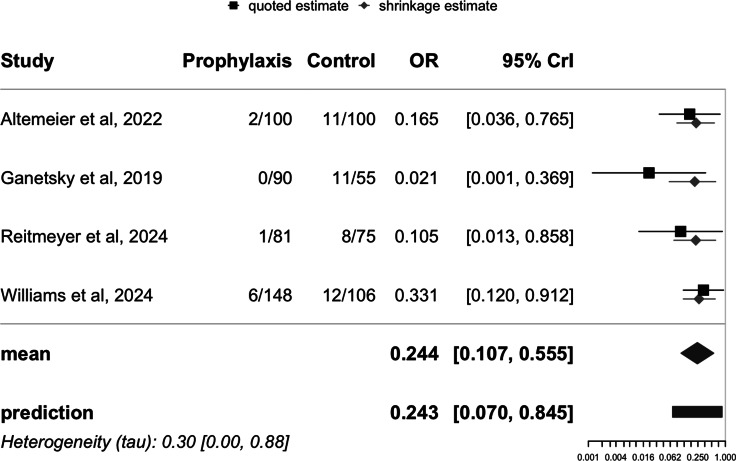
Forest plot of unadjusted *Clostridioides difficile* infection risk in stem cell transplant patients: impact of oral Vancomycin primary prophylaxis, full-text published studies only.

A Bayesian meta-analysis was conducted to estimate the pooled mean effect of the intervention across the six studies. Unadjusted analysis showed that oral vancomycin prophylaxis was associated with a reduced incidence of CDI, with a mean OR of .31 (95% CrI: .16 – .59), see Figure 2. A sensitivity analysis including only the four studies published in full text yielded similar results (mean OR: .24; 95% CrI: .11 – .56), see Figure 3.

Further analysis was conducted to estimate the overall mean effects of secondary outcomes across the studies, including bloodstream infections (OR 1.10; 95% CrI .65 – 1.81), positive vancomycin-resistant *Enterococcus* cultures (OR .75; 95% CrI .31 – 1.78), GVHD (OR 1.21; 95% CrI .59 – 2.47), and length of hospital stay (OR .90; 95% CrI .29 – 2.72). However, none of these assessments demonstrated a statistically significant effect (details available in the Supplementary Section).

A variety of secondary outcomes were studied: Four studies reported on bloodstream infections,^25–27,30^ while four also examined vancomycin-resistant *Enterococcus* isolated from any clinical culture.^25–27,29^ Three studies focused on GVHD.^25,26,29^ Lengths of hospital stay were reported in days by five studies; among these, two provided estimates of variability,^27,28^ two did not report variability estimates,^25,26^ and one study stratified length of stay by transplant type, including its variance.^30^ The remaining study did not mention length of hospitalization.^29^ Additionally, only two studies reported the use of systemic antibiotics during hospitalization,^26,28^ and two studies assessed event-free survival at one year.^25,26^ One study reported allergic reactions to vancomycin,^27^ and one study mentioned that patients with prior allergic reactions were excluded.^26^

### Quality assessment scores

In terms of quality assessment scores, one study was classified as good quality,^27^ while three studies were rated as fair quality,^25,26,28^ all of which were published in full text. By contrast, the two studies published only as abstracts were deemed poor quality according to the Downs and Black quality assessment tool (see Supplementary Table 2).^29,30^

### In-depth assessment of publication bias

**Figure 4.**
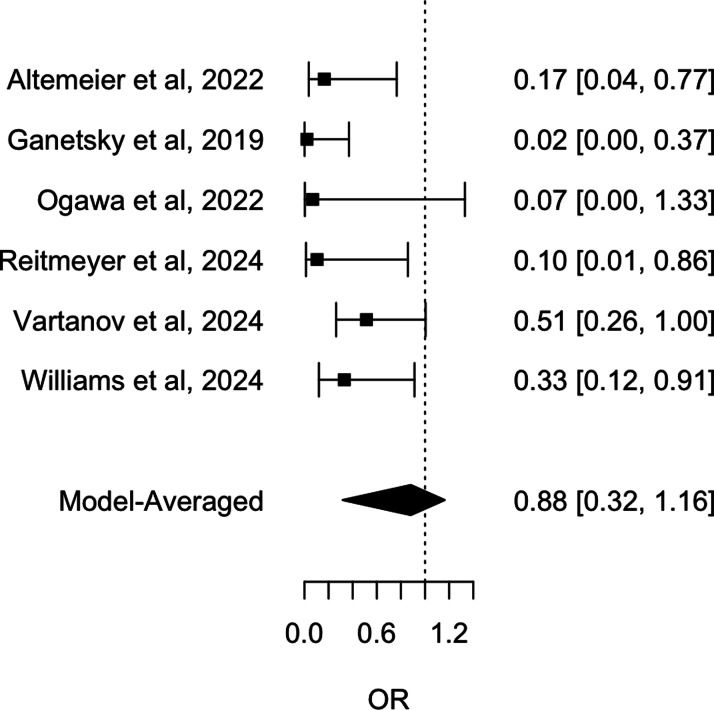
Forest plot of RoBMA-adjusted *Clostridioides difficile* infection risk in stem cell transplant patients: impact of oral Vancomycin primary prophylaxis.

**Figure 5.**
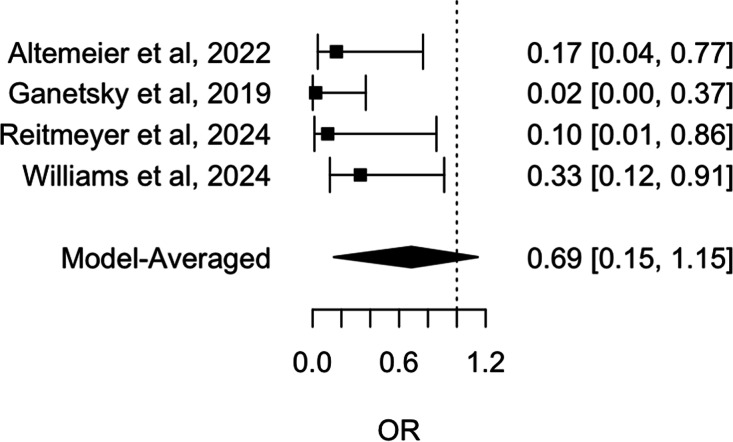
Forest plot of RoBMA-adjusted *Clostridioides difficile* infection risk in stem cell transplant patients: impact of oral Vancomycin primary prophylaxis, full-text published studies only.

The potential for publication bias was assessed using three methods. The funnel plot exhibited significant asymmetry, with a noticeable concentration of studies reporting protective effect sizes and a lack of smaller studies with opposing or null results. This pattern suggests the potential presence of publication bias, indicating that studies with less favorable outcomes may be underrepresented in the literature. Egger’s test confirmed this asymmetry (*P* = .001). Furthermore, RoBMA, a robust Bayesian model-averaged meta-regression analysis, found weak evidence of heterogeneity among the studies (BF = .68); and a strong suggestion of publication bias (BF = 97.70). We also employed RoBMA to simultaneously account for effect size, heterogeneity, and publication bias to estimate the overall effect of oral vancomycin prophylaxis. The RoBMA model found that the association between oral vancomycin prophylaxis and the incidence of CDI was statistically nonsignificant, with an OR of .88 (95% CrI: .32 – 1.16), therefore confirming the strong presence of publication bias, see Figure 4. A RoBMA sensitivity analysis including only the four studies published in full text yielded similar results (heterogeneity BF = .77 and publication bias BF = 12.25), see Figure 5.

## Discussion

This is one of the first meta-analyses addressing the use of oral vancomycin as primary prophylaxis specifically in the HSCT patient population. Oral vancomycin primary prophylaxis appeared to protect against the incidence of CDI. All secondary outcomes were no different after implementation of vancomycin prophylaxis (eg, vancomycin-resistant *Enterococcus*, GVHD). However, inherent characteristics of the included studies (eg, publication bias) prevented us from more strongly concluding that oral vancomycin should be used in this patient population.

We found that oral vancomycin primary prophylaxis is associated with preventing CDI in HSCT patients. While a meta-analysis of general inpatient populations found primary oral vancomycin prophylaxis ineffective,^31^ two meta-analysis including immunocompromised transplant patients showed it to be beneficial.^32,33^ The American College of Gastroenterology endorses oral vancomycin secondary prophylaxis,^34^ but the Infectious Disease Society of America’s latest guidelines do not address CDI prophylaxis.^35^ Furthermore, the European Society of Clinical Microbiology and Infectious Diseases do not recommend the routine use of oral vancomycin prophylaxis.^36^ Our meta-analysis uniquely examines the HSCT population without known history of prior CDI, providing new insights into the efficacy of oral vancomycin prophylaxis in this high-risk group.

Oral vancomycin as primary prophylaxis did not change the incidence of vancomycin-resistant *Enterococcus* or bloodstream infections in this population. It is a rational choice for CDI prophylaxis due to its strong efficacy against *C. difficile*, minimal long-term systemic adverse effects, and cost-effectiveness.^37^ However, it alters the gut microbiome,^38^ potentially increasing the risk of colonization by vancomycin-resistant *Enterococcus* and other multidrug-resistant organisms. While studies in this meta-analysis showed no impact on vancomycin-resistant *Enterococcus* positivity during transplant hospitalization, other studies also found no significant differences in infection rates with or without oral vancomycin secondary prophylaxis.^31–33^ Consequently, the long-term effects of oral vancomycin on the colonization of vancomycin-resistant *Enterococcus* or other multidrug-resistant organisms remain uncertain.

Similarly, we found no significant differences in the length of hospitalization or the incidence of GVHD between HSCT patients who received primary prophylaxis and those who did not. Despite the established association between prolonged hospital stays with increased incidence of CDI,^4^ the length of hospital stay remained consistent before and after the implementation of oral vancomycin prophylaxis. Although it has been hypothesized that CDI may trigger gastrointestinal GVHD,^39^ the reduced incidence of CDI in the prophylaxis group did not translate into a measurable impact on GVHD rates. Notably, these secondary outcomes were not evaluated in previous studies with similar objectives.

Our analysis revealed a significant publication bias, prompting us to conduct a robust Bayesian model-averaged meta-analysis. While meta-analyses are valuable tools for integrating data and informing decision-making in evidence-based medicine, publication bias remains a major limitation,^40^ as it can lead to an overestimation of intervention effects. To address this issue, we employed a statistical method that tests and adjusts for effect size, heterogeneity, and publication bias.^23^ RoBMA is an innovative methodological approach that integrates these factors into an adjusted model, which is relatively new in this field. Although our unadjusted analysis indicated a significant association between oral vancomycin prophylaxis and reduced odds of CDI, the publication bias-adjusted model revealed a nonsignificant effect of primary prophylaxis. This underscores the urgent need for randomized controlled trials to provide more robust evidence regarding this association.

This systematic review has additional limitations that should be considered when interpreting the findings. First, all included studies were observational in nature, which may introduce biases, including confounding factors that could influence the estimated impact of oral vancomycin prophylaxis on the incidence of CDI. Additionally, variability in patient populations and diagnostic protocols for CDI across the studies may limit the generalizability of the results. The quality assessment of the included studies identified several methodological weaknesses. Furthermore, the limited number of studies and the small sample sizes within each study included in the meta-analysis may restrict statistical power and the ability to detect subtle differences in outcomes.

In conclusion, this systematic review and Bayesian meta-analysis indicate that there is weak evidence in favor of primary oral vancomycin prophylaxis in preventing CDI in patients undergoing HSCT, and the current data show no immediate indications of harm. Given the heightened vulnerability of this patient population to CDI and the potential complications that can arise, such as GVHD and non-relapse mortality, oral vancomycin prophylaxis emerges as a potential intervention that merits further investigation. Nonetheless, critical questions remain regarding its long-term safety, potential effects on antimicrobial resistance, influence on the gut microbiome, and its efficacy in patients with a history of CDI or colonization. Addressing these questions in future research will be essential to fully elucidate the benefits and risks of oral vancomycin prophylaxis in this high-risk population.

## Supporting information

10.1017/ash.2025.10179.sm001 Viana-Cardenas et al. supplementary material Viana-Cardenas et al. supplementary material

## Supplementary material

The supplementary material for this article can be found at https://doi.org/10.1017/ash.2025.10179.
